# Egg exosome miR-145-5p decreases mitochondrial ROS to protect chicken embryo hepatocytes against apoptosis through targeting *MAPK10*

**DOI:** 10.1186/s40104-025-01203-y

**Published:** 2025-05-24

**Authors:** Fengdong Zhang, Yongchang Han, Fan Li, Boya Guo, Jian Chen, Wenchuan Zhou, Pan Xiao, Hui Ma, Yongyan Jin, Jia Feng, Yuna Min

**Affiliations:** College of Animal Science and Technology, Northwest A&F University, Shaanxi, 712100 China

**Keywords:** Aged breeding hen, Apoptosis, Exosome, Hatchability, MiR-145-5p

## Abstract

**Background:**

Higher embryonic mortality, especially in aged breeding hens, is associated with insufficient hepatic functionality in maintaining redox homeostasis. Our previous study demonstrated that egg exosome-derived miRNAs may play a key role in modulating embryonic oxidation-reduction process, whereas the exact function and mechanism were still poorly understood. The present study aimed to investigate the roles of egg exosome miRNAs in maintaining dynamic equilibrium of free radicals and peroxide agents in embryonic liver, as well as demonstrate the specific mechanism using oxidative stress-challenged hepatocytes.

**Results:**

Compared to 36-week-old breeding hens, decreased hatchability and increased embryonic mortality were observed in 65-week-old breeding hens. Meanwhile, the older group showed the increased MDA levels and decreased SOD and GSH-Px activities in embryonic liver, muscle and serum. Embryonic mortality was significantly positively correlated with MDA level and negatively correlated with GSH-Px activity in embryonic liver. In addition, 363 differentially expressed genes (DEGs) were identified in embryonic liver, 13 differentially expressed miRNAs (DE-miRNAs) were identified in egg exosomes. These DEGs and DE-miRNAs were involved in oxidoreductase activity, glutathione metabolic process, MAPK signaling pathway, apoptosis and autophagy. miRNA-mRNA network analysis further found that DEGs targeted by DE-miRNAs were mainly enriched in programmed cell death, such as apoptosis and autophagy. Wherein, *MAPK10* with highest MCC and AUC values was significantly related to GSH-Px activity and MDA level, and served as the target gene of miR-145-5p based on dual luciferase reporter experiment and correlation analysis. Bioinformatics analysis found that miR-145-5p/*MAPK10* axis might alleviate peroxide generation and apoptosis. In primary hepatocytes of chick embryos, miR-145-5p transfection significantly reversed H_2_O_2_-induced mitochondrial ROS increase, *MAPK10*, *BAX* and *CASP3* overexpression and excessive apoptosis.

**Conclusion:**

Exosome miR-145-5p in eggs could target *MAPK10* and decrease mitochondrial ROS, attenuating oxidative damage and apoptosis in hepatocytes of chick embryos. These findings may provide new theoretical basis for the improvement of maternal physiological status to maintain embryonic redox homeostasis by nutritional or genetic modifications.

**Graphical Abstract:**

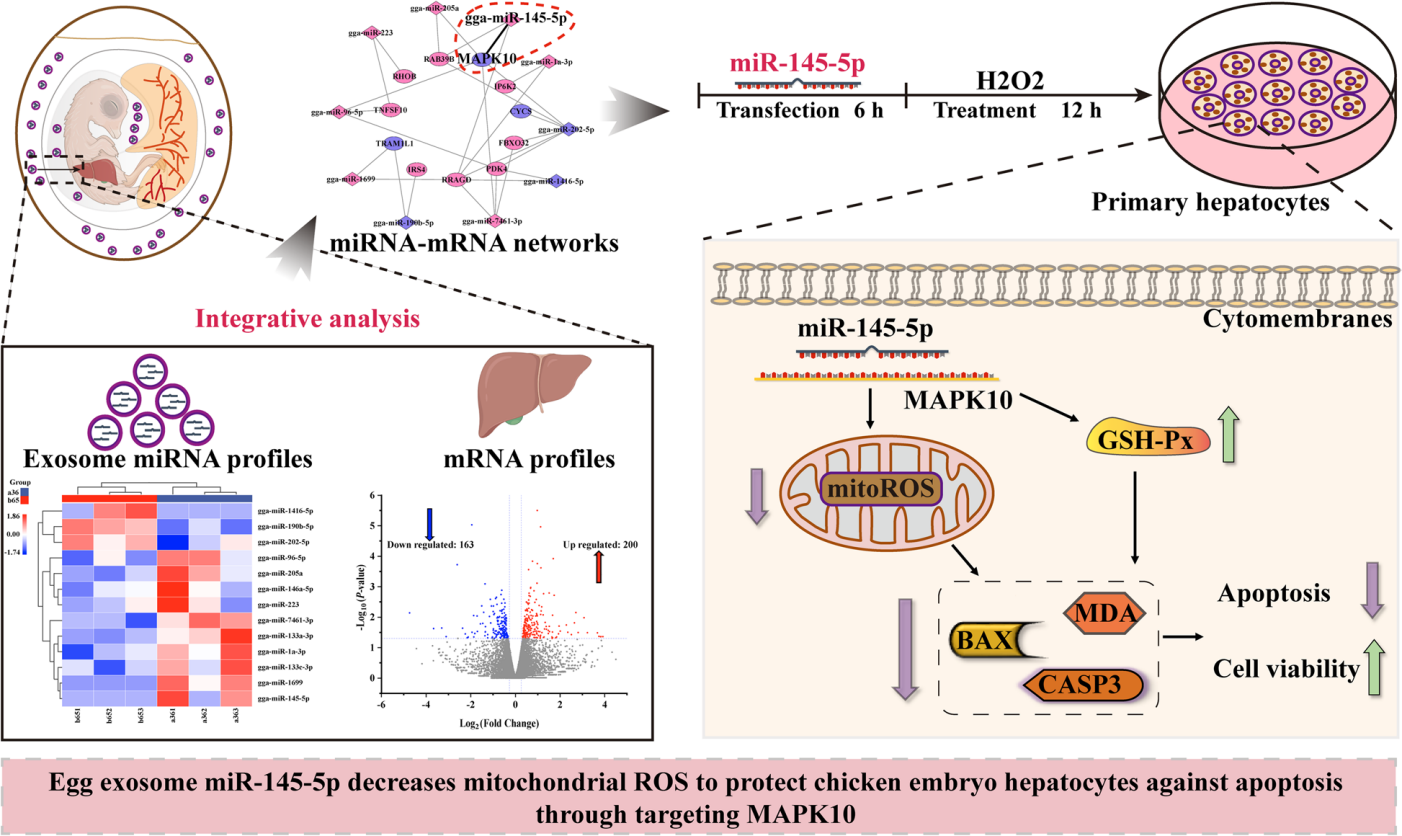

**Supplementary Information:**

The online version contains supplementary material available at 10.1186/s40104-025-01203-y.

## Background

Declined hatchability in the late laying phase, reduced by approximately 6% compare to peak laying period, greatly limits implementation for extending laying period of breeding hens, compromising the economic benefits of farmers [1]. It was found that the increased embryonic malformation and mortality could be attributed to the exacerbated oxidative damage of chick embryos [2]. Embryonic redox homeostasis and development are closely related to biomolecules from maternal sources [3–5]. Pro-oxidative molecules in aged breeding hens would be easily transferred into fertile eggs aggravating embryonic oxidative damage [6, 7]. Hence, exploring effects and mechanisms of key biomolecules in eggs to embryonic oxidative damage are of great significance for increasing the hatching performance of aged breeding hens.

Exosomes are crucial mediators transmitting communication signals of the embryonic-maternal cross-talk, and contain many genetic molecules that play key roles in fetal development, physiological and pathological process, like miRNAs and mRNAs [8]. Recently, exosome miRNAs derived from amniotic fluid were demonstrated to take a significant role in maintaining redox homeostasis and attenuating inflammatory responses [9]. Administration of exosome miR-17-3p from amniotic fluid depressed reactive oxygen species (ROS) contents and increased GSH-Px activity in serum, thus alleviating inflammatory reaction of mice during oxidative stress [10]. In poultry, it was found that egg exosome miRNAs could participate in oxidation–reduction processes, implying their roles in maintaining dynamic balance of ROS in chick embryos [11–13]. However, these exosome miRNAs may play opposite roles in regulating oxidative stress, for example, some miRNAs could alleviate oxidative stress-induced apoptosis, while others could exacerbate ROS generation and cellular structure damage [14–17]. Actually, exosomal miRNA profiles and function are attributed to the physiological state of parental cells [18]. Senescence or oxidative stress could significantly alter miRNA profiles in exosomes, leading to oxidative imbalance of recipient cells like endogenous anti-oxidant system destruction and ROS accumulation, and even promoting apoptosis [18, 19]. Changes of miRNAs profiles have also been identified in reproductive system of aged hens, in which many miRNAs could increase H_2_O_2_-induced cell death [20–22]. Biomolecules in reproductive system of laying hens could be transferred into fertile eggs, participating in embryonic oxidation–reduction balance and development [2, 7]. Thus, it could be speculated that egg exosome miRNAs in aged breeding hens might exacerbate embryonic oxidative damage. Significantly, effects of miRNAs are attributed to their target genes and corresponding cascade reactions including autophagy, apoptosis, glutathione metabolism and oxidoreductase activity pathways [23–26]. However, it is still unclear the regulative effects of exosome miRNAs in redox balance of chick embryos.

This study aimed to investigate the potential mechanism of peroxide accumulation in embryonic liver of aged breeding hens from the perspective of exosome miRNAs. Correlation analysis and random forest model revealed the relationship between endogenous antioxidant activities, peroxide agent levels and DEGs abundance in embryonic liver. Then, exosome DE-miRNAs, miRNA-mRNA networks and corresponding signaling pathways were disclosed based on high throughput sequencing analysis. Effects and regulative mechanism of candidate miR-145-5p/*MAPK10* axis in oxidative damage were investigated using H_2_O_2_-challenged primary hepatocytes of chick embryos. Our findings would shed light on mechanism that exosome miRNAs regulate liver oxidative damage of chick embryos, which provide theoretical basis and guidance for maintaining embryonic redox homeostasis by improving maternal physiological status through nutritional or genetic modifications.

## Materials and methods

### Incubation of egg

A total of 528 fertilized eggs from the 36-week and the 65-week Hy-Line Brown hens were obtained from Ningxia Xiaoming Group Co., Ltd. (Ningxia, China). Eggs in each group were divided homogeneously into 6 replicates (44 eggs on a tray) and incubated in the automatic-controlled incubator maintaining at 37 ± 0.1 °C and 60% relative humidity. Fertilized eggs with similar weight were selected to incubation, for avoiding the influence of egg weight on hatching performance. At 7 and 14 d of incubation, nonviable and unfertilized eggs were identified by candling inspection and taken out of incubator. Subsequently, eggs were transferred to hatching baskets at 18 d of incubation and went on hatching process at 37.2 ± 0.1 °C and 72% relative humidity. The number of dead embryo at the pre-, mid- and post-incubation periods was recorded and used to calculate the embryonic mortality. At d 17, 18, 19 and hatch, yolk and yolk-free body weight were recorded for determining the rate of yolk absorption. From 480 h (d 20) of incubation, the number of chicks in each replicate was recorded at 3 h intervals and used to calculate the hatch window period. At hatch, chicks were weighed and counted to calculate average body weight and hatching rate. Chick quality was determined with the scoring system proposed in previous study [27].

### Sample collection and biochemical analyses

On d 18 and 19 during incubation, blood, liver and muscle were harvested from embryo (3 embryo/replication) and stored at −80 °C. Egg exosomes were obtained according to our prior report [11]. Commercial kits (Nanjing Jiancheng Bio-Engineering Institute, Nanjing, China) was selected to measure biochemical indicators, including activities of glutathione peroxidase (GSH-Px), catalase (CAT) and superoxide dismutase (SOD), total antioxidant capacity (T-AOC) and levels of protein and malondialdehyde (MDA).

### mRNA-seq and bioinformatics analysis

Total RNAs were acquired using TRIzol reagent (Invitrogen, Carlsbad, CA, USA). Enrichment and purification were executed to remove ribosomal rRNAs by magnetic beads with Oligo(dT) for obtaining more effective information. Then, cDNA was synthesized using random primers with fragmented RNAs as the template. Entire sequence libraries were prepared by PCR amplification after screening and recovering target fragments using magnetic beads. The quality of sequence library was determined by Agilent 2100 Bioanalyzer (Agilent Technologies Inc., California, USA). The concentration of sequence library was determined by qPCR analysis. These original sequence were filtered to eliminate contaminated reads with low-quality after sequencing the qualified library using Illumina platform (Personalbio, Shanghai, China). The genome indexing was acted using GRCg6a as the reference FASTA and the Ensembl Gene annotation file (Gallus_gallus.GRCg6a.dna.toplevel.fa; http://ftp.ensembl.org/pub/release-105/fasta/gallus_gallus/dna). Subsequently, we measured gene expression levels and normalized the transcriptome count matrix, which were shown as Fragments Per Kilobase of script per Million fragments mapped (FPKM). According to the screening criteria *P*_adjust_ < 0.05 and |log_2_Fold Change| > 1, differentially expressed genes (DEGs) between 36-week and 65-week groups were screened. Biological functions of DEGs were disclosed using Gene Ontology annotation (GO), which were mainly divided into molecular functions (MF), cellular components (CC) and biological processes (BP). Meanwhile, Kyoto Encyclopedia of Genes and Genomes (KEGG) pathway analysis was acted to reveal noteworthy pathways. These analyses, GO and KEGG, were performed by the software of Pathway network (https://www.pathwaysnetwork.net).

### Small RNA sequencing analysis and miRNA identification

After acquiring total RNAs, sequencing libraries were prepared using TruSeq Small RNA Sample Prep Kits (Illumina, San Diego, USA) and detected on Illumina Hiseq2500/2000. Firstly, 3' adapter and 5' adapter were successively ligated to the end of 3' or 5' of these miRNAs. After that, cDNA was synthesized by reverse transcription using reverse transcriptase for preparing cDNA libraries. When PCR amplification had been performed, products were purified on 6% Novex TBE PAGE gel. These DNA fragments with range from 145 to 160 bp were recovered into a 2-mL tube. After concentrating by ethanol precipitation, Agilent Bioanalyzer 2100 system with DNA-1000 chips was used to examine the size, concentration and purity of these samples. To identify known miRNAs, clean tags acted comparison in miRBase database (Release 22). Finally, miRNAs were analyzed using these softwares, including ACGT101-miR (https://www.bioz.com/search?q=acgt101%20mir%20v4%202&other=true&exact_search=true&rating_sort=true&dtx=1), TargetScan (https://www.targetscan.org/vert_80) and Pathway network (https://www.pathwaysnetwork.net/).

### Construction of miRNA-mRNA Network

Target genes of differential miRNAs conducted comparative analysis with differentially expressed mRNAs identified by transcriptome. These overlapping mRNAs were selected to construct the miRNA-mRNA networks. Analysis of miRNA-mRNA networks was acted using Cytoscape (v3.9.1).

### Protein–protein interaction analysis

The protein–protein interaction (PPI) network was constructed by string database (https://cn.string-db.org). Wherein, degrees of all nodes were calculated using topological analyses. Visualization of PPI network was performed with Cytoscape v3.9.1. Finally, hub genes were identified using cytoHubba. CytoHubba is a Cytoscape plug-in and commonly used to explore the network modules for identifying hub genes.

### miRNA transfection

Primary hepatocytes were isolated from embryonic liver at d 18 of incubation (E18), as described previously [28]. Hepatocytes were seeded in 6-well plates with a density of 2 × 10^5^ cells/well and cultured for 48 h. Subsequently, medium in plates was replaced with fresh culture medium that did not contain penicillin/streptomycin, and plates were then cultured for 3 h. Mimics and inhibitors of miR-145 (20 nmol/L) were respectively transfected into cells by Lipofectamine 2000. Here, miR-NC means negative control fragments; mi-miR-145 means mimics of miR-145; in-miR-145 means inhibitors of miR-145. Following 6 h of transfection, medium with H_2_O_2_ (1 mmol/L) or not were used to replace medium in plates and continuously cultured for 12 h. All miRNA mimic and inhibitor were chemically synthesized by GenePharma, Co., Ltd. (Shanghai, China).

### Quantitative real-time PCR assays

Total RNAs in liver tissues and cells were extracted using TRIzol reagent (Invitrogen, Carlsbad, CA, USA) according to the manufacture’s protocols. The concentration and purity of total RNAs were detected by agarose gel electrophoresis and microplate reader respectively. Then, cDNA was synthesized by reverse transcription using PrimeScript RT Reagent kits (Genenode, Wuhan, China). Quantitative analysis was performed on a QuantStudio Real-Time PCR System (Thermo Fisher Scientific, MA, USA) using SYBR Green reagent kits (Genenode). Primers were synthesized by Sangon Biotech Co., Ltd. (Shanghai, China) and listed in Table S1. Calculation of relative expression was same with previously described 2^−ΔΔCt^ method [29].

### Apoptosis measurement

Apoptotic rates of cells were examined by Annexin V-FITC/PI kits (4 A Biotech, Suzhou, China). After staining with Annexin V-FITC and PI following manufacture’s requirement, suspension of these cell samples were measured using flow cytometry system.

### Cell viability

Cell viability was tested with cell counting kits-8 (CCK8, Beyotime, Shanghai, China). In brief, 10 μL CCK8 reagent was added into each well incubated for 3 h, after finishing all experimental treatment. Subsequently, a microplate reader (Thermo Fisher Scientific) was used to measure the absorbance of each well at 450 nm.

### Measurement of mitochondrial ROS

Mitochondrial ROS was detected by MitoSOX Red probes (MedChemExpress, Shanghai, China). After treatment, hepatocytes were washed with PBS, digested with trypsin and re-suspend with PBS. Then, 5 μmol/L MitoSOX Red probes were added into hepatocytes, staining for 30 min at room temperature. After centrifugation (400 × *g*, 5 min), hepatocytes were washed twice using PBS. Finally, hepatocytes were detected at excitation/emission wavelengths of 510 nm/590 nm using flow cytometry, fluorescence microscope and microplate reader.

### Dual-luciferase reporter assay

Cells were seeded in 6-well plates at a density of 2.5 × 10^5^ cells/well. Sequence of *MAPK10* as wild type (WT) and mutation type (MT) were cloned into the pmirGLO Vector. These pmirGLO Vector and miR-145 mimics were co-transfected into cells using Lipofectamine 2000. Using a Dual-Luciferase Reporter Assay System (Promega Corporation) to detect luciferases activity following cultivation of 48 h.

### Statistical analysis

Data in the current study was analyzed using SAS 9.4 and presented as mean ± standard deviation (SD). Differences of these data were tested by *t*-test (two groups) and one-way analysis of variance (ANOVA) with a Duncan’s post hoc test (three or more groups). GraphPad Prism software 8.0 (GraphPad, La Jolla, CA, USA) generated graphical data. Pearson's correlation coefficient analyzed correlations between antioxidant capacities, hatchability, embryonic mortality and expression of mRNAs. Statistical difference with *P* < 0.05 was applied.

## Results

### Differential hatching performance between young and aged breeder flock

**Fig. 1:**
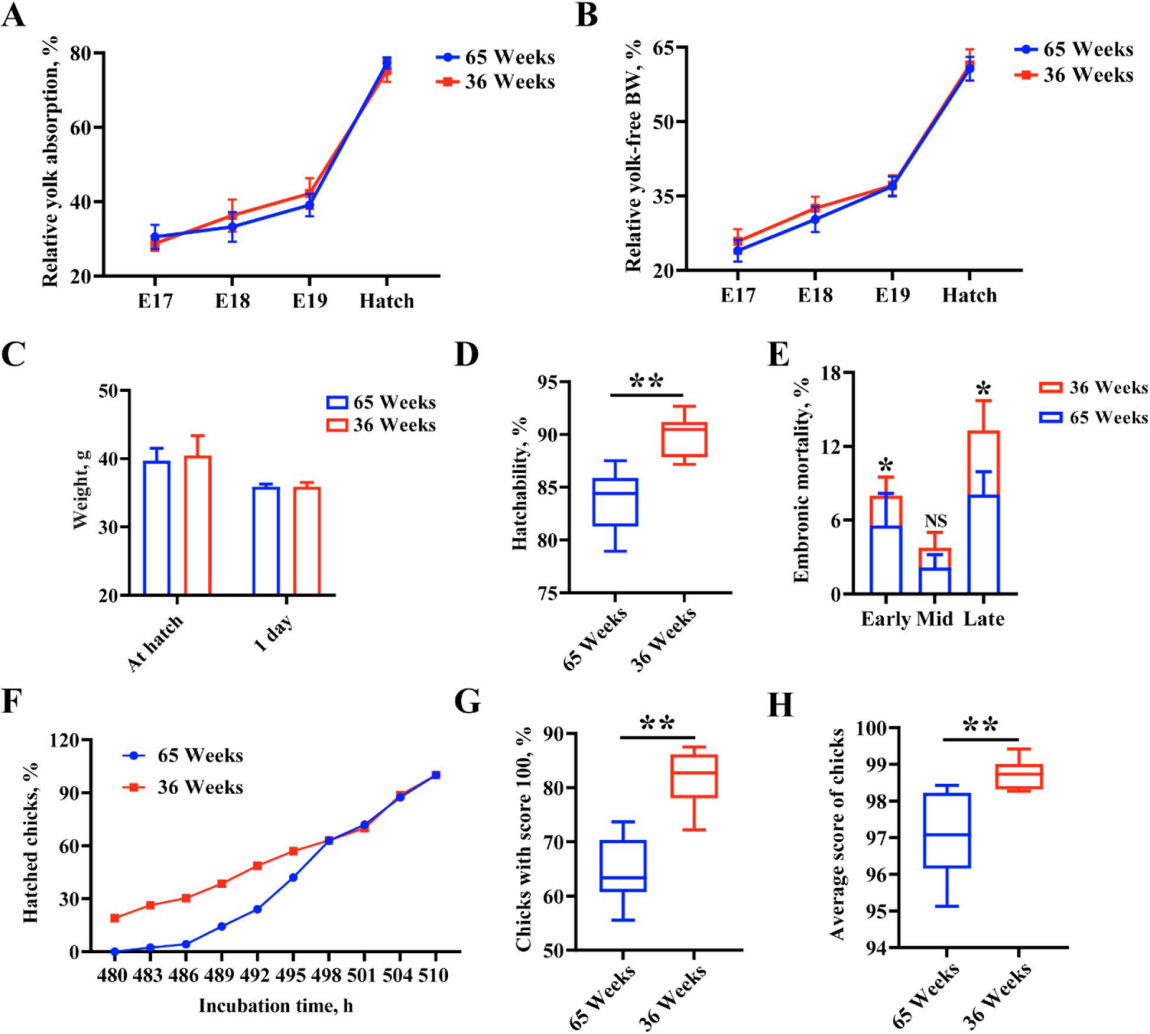
Differential analysis of hatching performance in breeding hens at 36 and 65 weeks. **A** and **B** Relative yolk absorption and yolk-free WB determined in different time of incubation. **C** Chick weight were identified at hatch and 1 d. **D** and **E** Hatchability and embryonic mortality (at early, middle and late period of incubation). **F** Hatch window in breeding hen aged 36 and 65 weeks. **G** and **H** Chick quality was assessed with these data such as chicks with score 100 and average score of chicks. ^*^P < 0.05, ^**^P < 0.01

As shown in Fig. S1, compared to 36-week group, initial weights of eggs, albumen and yolk were not significant difference, however, dry weight of albumen and yolk:albumen ratio exhibited significantly different in 65-week group. Significant differences in relative yolk absorption and relative yolk-free BW among groups were not observed at E17, E18, E19 and hatch (Fig. 1A and B). In addition, there were not significantly different in BW of chicks at hatch and 1 day (Fig. 1C). It was noteworthy that increased hatchability, decreased early and late embryonic mortality in 36-week group were observed, compared to 65-week group (Fig. 1D and E). Spread of hatch was not observed significant difference between these two groups (Fig. 1F). The score of chicks (chicks with score 100 and average score of chicks) in 36-week group were significantly higher than that in 65-week group (Fig. 1G and H). In one word, hatchability and chick quality from 36-week-old breeding hens were better than 65-week-old breeding hens.

### Antioxidant enzyme activity and correlation analysis

**Fig. 2:**
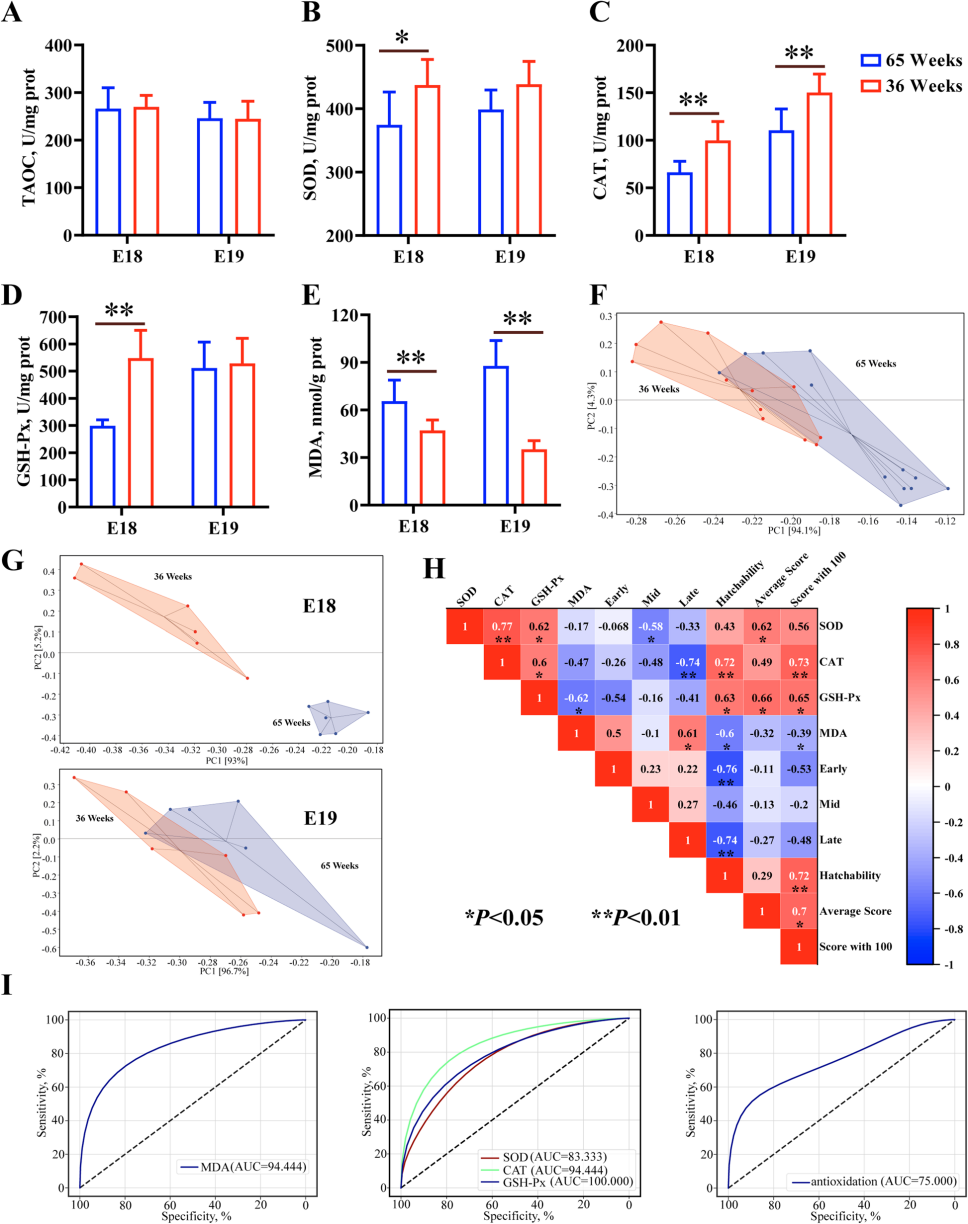
Differential analysis of antioxidant capacities in embryonic liver. **A–E** T-AOC, SOD, CAT and GSH-Px activities and MDA levels in liver of embryos at E18 and E19. **F** and **G** Principal component analysis (PCA) was performed for identifying the difference of antioxidant capacity of embryonic liver. **H** Overview of correlation analysis. **I** Based on the random forest model, classification of hatchability using antioxidant capacity related data. ^*^P < 0.05, ^**^P < 0.01

Compromised antioxidant capacity of embryos might be one of the important reasons to the lower hatching performance [30]. Here, we performed differential analysis to SOD, GSH-Px, CAT and MDA in embryos from eggs from 36-week-old breeder flocks (36-Em) and 65-week-old breeder flocks (65-Em). At d 18 embryonic period (E18), compared with 36-Em, the activities of SOD, CAT and GSH-Px decreased significantly, the level of MDA increased significantly in 65-Em liver (Fig. 2A–E). At d 19 embryonic period (E19), compared with 36-Em, CAT activity decreased significantly, MDA level increased significantly in 65-Em liver (Fig. 2A–E). The antioxidant capacity of serum and muscle, but not the liver, in 36-Em was stronger than that in 65-Em, as evidenced by the increased activities of SOD and GSH-Px and decreased level of MDA (Fig. S2). Liver, a central metabolic organ, play key roles in maintaining redox homeostasis [31]. Using principal component analysis, remarkable differences were observed in antioxidant capacity of liver between 36-Em and 65-Em (Fig. 2F), especially at E18 (Fig. 2G). Thus, data at E18, including SOD, CAT and GSH-Px activities and MDA level, was conducted correlation analysis with hatching performance. Analysis of spearman correlations showed that hatchability was positively correlated with CAT and GSH-Px activities and negatively correlated with MDA level and embryonic mortality, embryonic mortality was positively correlated with MDA level and negatively correlated with CAT activity (Fig. 2H). Notably, random forest model revealed that GSH-Px, CAT, MDA and SOD had AUC values of 1, 0.94, 0.94 and 0.83 in classified hatchability, indicating that hatchability was closely related to antioxidant capacity of embryonic liver (Fig. 2I).

### Differential expression profiles of mRNAs in embryonic liver

**Fig. 3:**
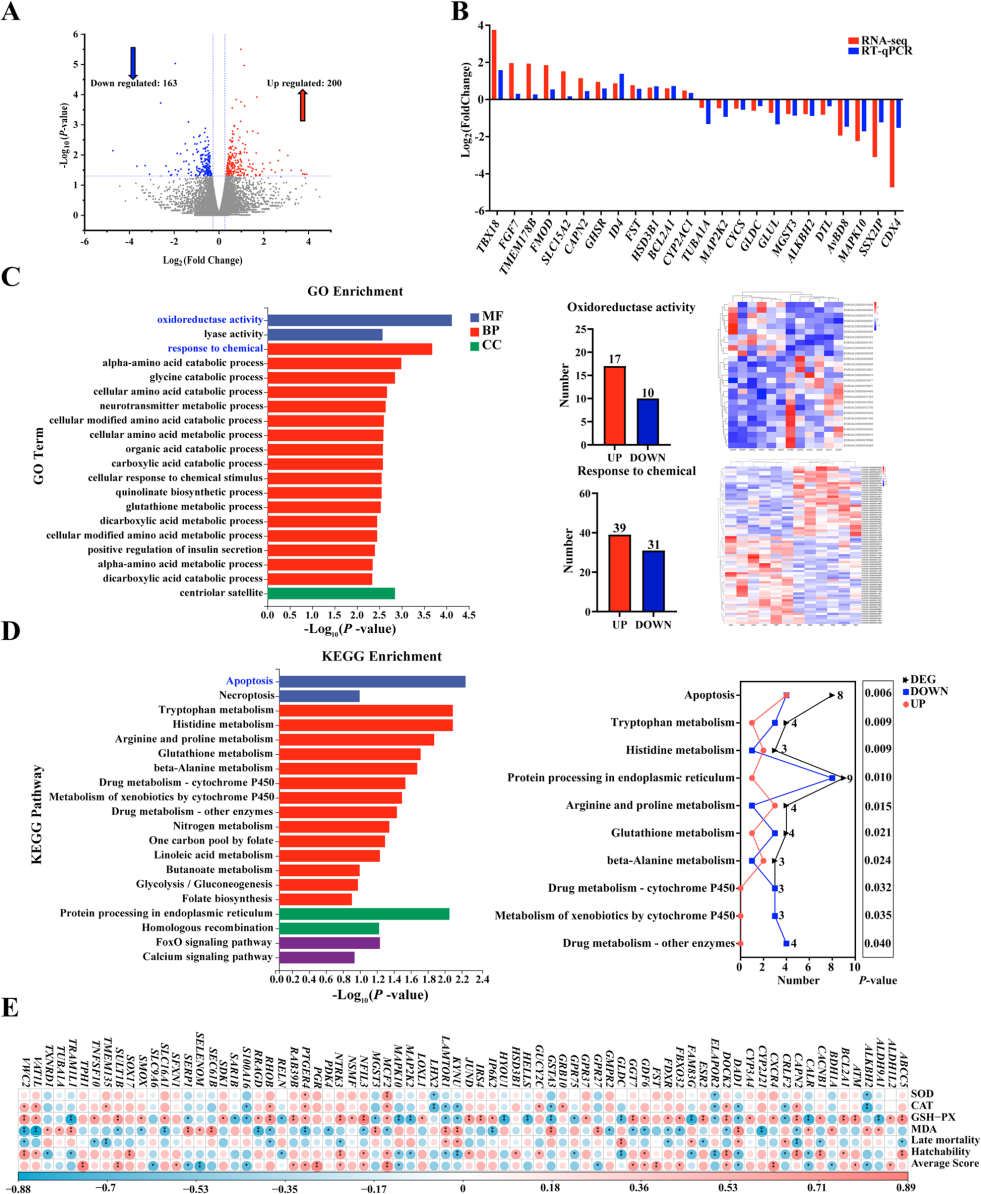
Expression profiles and functional analysis of mRNAs in embryonic liver from 36 and 65 week-old breeding hens. **A** Volcano plots displaying differentially expressed mRNAs. **B** The 24 differential mRNAs obtained by RNA-seq were validated using qRT-PCR. **C** and **D** Gene Ontology (GO) functional enrichment analysis and Kyoto Encyclopedia of Genes and Genomes (KEGG) enrichment analysis. **E** Overview of correlation analysis. ^*^P < 0.05, ^**^P < 0.01

Transcriptomic analysis was used to explore the differential genes and key signaling pathways in 36-Em and 65-Em liver, for deeply understanding the mechanism that cause excessive accumulation of pro-oxidant molecules. As present in Fig. 3A, 200 DEGs were up-regulated, and 163 DEGs were down-regulated in 36-Em compared to 65-Em (Fig. 3A). Fold changes of DEGs within > 1.5 and < 0.5 were 80.44% (Fig. S3A). Meanwhile, DEGs had good repeatability within groups respectively according to cluster analysis and RT-qPCR (Fig. 3B and Fig. S3B). Subsequently, these 363 DEGs were mainly involved in regulating oxidation and stimulus reactions, such as oxidoreductase activity, glutathione metabolic process, response to chemical, cellular response to chemical stimulus, etc. (Fig. 3C). According to KEGG analysis, 10 significantly different KEGG pathways were identified, most of which were related to programmed cell death (apoptosis), oxidative stress (glutathione metabolism), protein processing (protein processing in endoplasmic reticulum), etc. (Fig. 3D). Among them, most of DEGs were enriched into apoptosis and protein processing in endoplasmic reticulum pathways (Fig. 3D). Pathways, such as programmed cell death, response to stimuli and protein-misfolding, etc., play key roles in redox homeostasis maintenance and have also aroused wide attention in alleviating pathological processes induced by oxidative stress [32–34]. According to correlation analysis, a large number of DEGs in these pathways were significantly related to hatchability, average score and GSH-Px activity (Fig. 3E and Fig. S3C), including those that were identified as the hub genes by PPI network analysis, such as *GSTA3*, *SEC61B*, *GLDC*, *MAPK10*, etc. (Fig. S3D).

### Differential expression profiles of miRNAs in egg exosomes

**Fig. 4:**
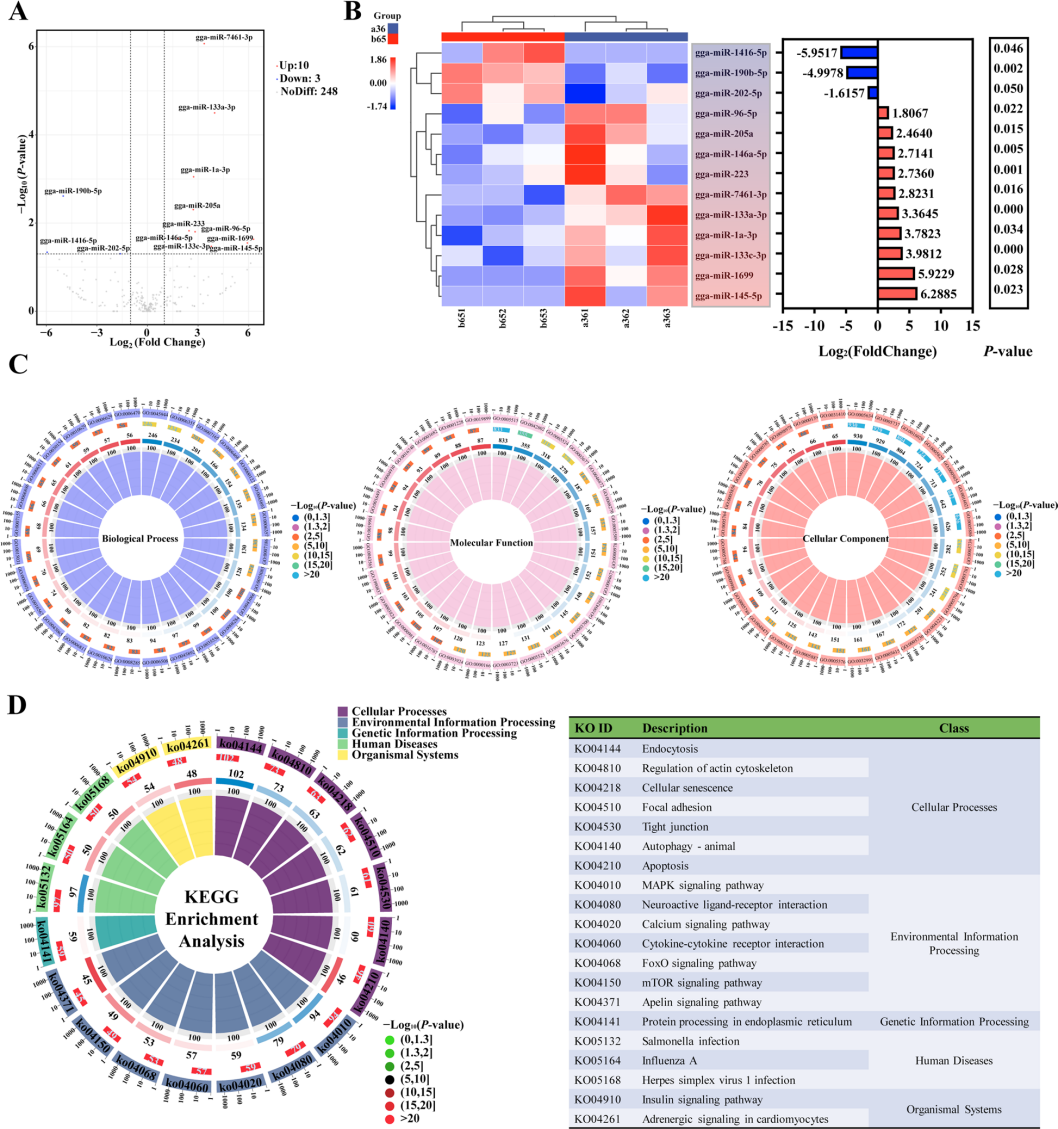
Expression profiles and functional analysis of egg exosome miRNAs. **A** and **B** Volcano plots and heatmap displaying differentially expressed mRNAs. **C** and **D** Gene Ontology (GO) functional enrichment analysis and Kyoto Encyclopedia of Genes and Genomes (KEGG) enrichment analysis

Our previous study found that egg exosome miRNAs could participate in redox processes and apoptosis [11]. In the present study, differences of exosome miRNAs in eggs from 36-week-old breeder flocks (36-Eg) and 36-week-old breeder flocks (65-Eg) were examined by high throughput sequencing combined with bioinformatics analysis. According to Counts Per Million (CPM), miRNAs exhibited various expressions across all samples (Fig. S4A). A clear difference of miRNA profiles was observed in two groups using principal component analysis (Fig. S4B). The sequence length of all miRNAs was distributed in 19–27 nt, among which 21, 22 and 23 nt miRNAs were most abundant (Fig. S4C). There were 13 differential expression miRNAs (10 up-regulated and 3 down-regulated miRNAs, DE-miRNAs) in two groups (Fig. 4A and B). Moreover, |log_2_(fold change)| values of these DE-miRNAs were all higher than 1 (Fig. 4B). To further understanding the function of 13 miRNAs, target genes of these miRNAs predicted by Miranda software were performed GO annotation and KEGG enrichment analysis. As shown in Fig. 4C and Table S2, signal transduction, transcription, protein modification and cellular processes including apoptosis, differentiation and proliferation were significant enrichment in the GO term biological process. In the molecular function category, these target genes were mainly related to binding, catalytic activity and oxidoreductase activity (Fig. 4C and Table S2). Cellular component included nucleus, cytoplasm, plasma membrane, cytoplasmic vesicle and mitochondrion (Fig. 4C and Table S2). According to KEGG enrichment analysis, these miRNAs were mainly related to endocytosis, cellular senescence, autophagy-animal, apoptosis and MAPK signaling pathway (Fig. 4D). In addition, over 15% of target genes of each miRNAs were involved in cellular processes and redox balance (Fig. S4D and E). According to PPI analysis, there were 5 hub genes in these target genes, including *MAPK3*, *PIK3CA*, *MAPK10*, *PRKACB*, *RAF1* (Fig. S4F). Wherein, *MAPK10*, *MAPK3* and *RAF1* might be the vital target genes of these miRNAs to function, based on PPI analysis, targeting relationship and signaling pathway analysis (Fig. S4G). Based on these results, it could be inferred that egg exosome miRNAs possibly exhibit pivotal effects in cellular processes and redox balance of chick embryos, but further analyses were necessary to reveal the vital network pathways.

### Integrative analysis of miRNA and mRNA expression profiles

**Fig. 5:**
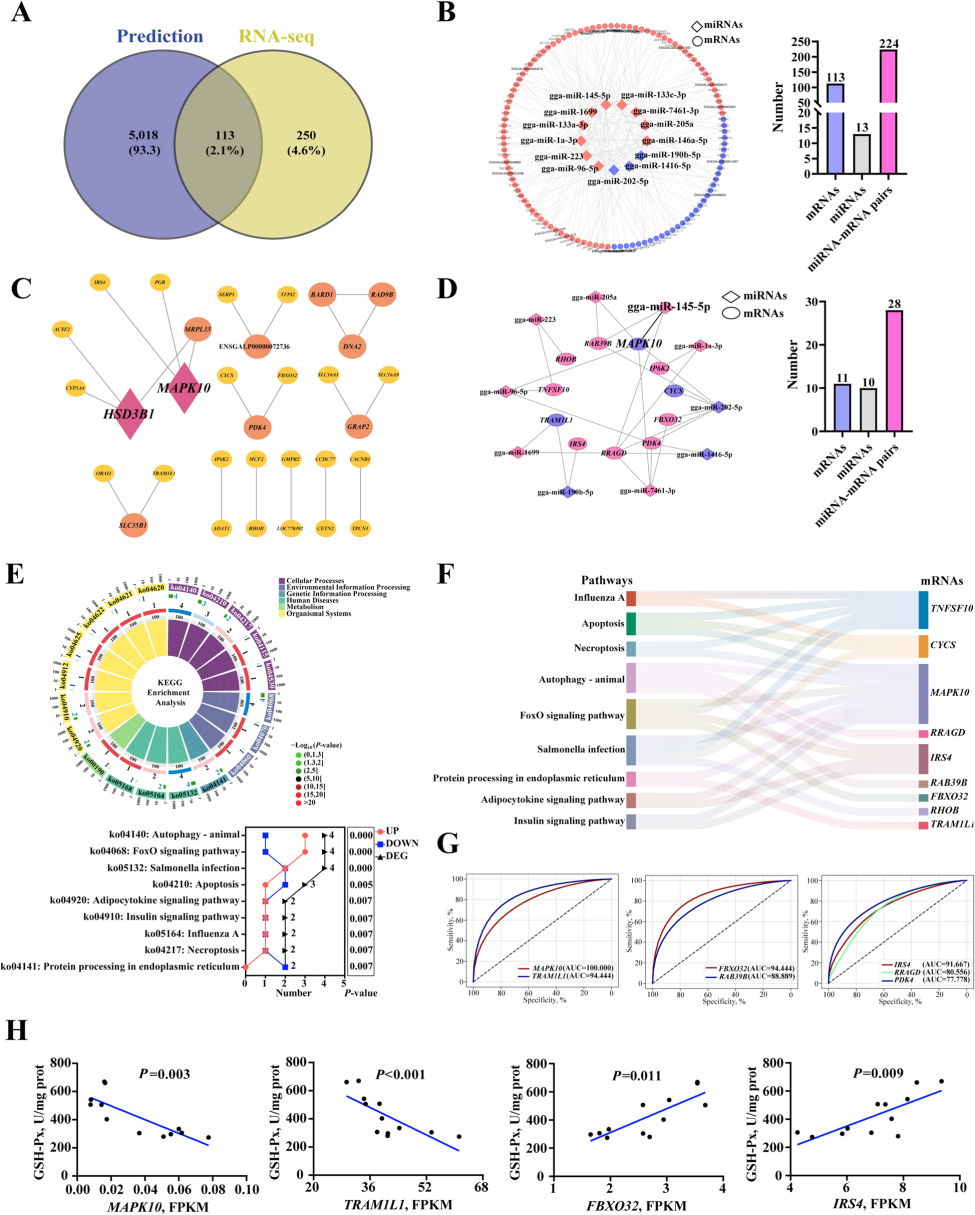
Construction of the miRNA-mRNA network by integrative analysis. **A** Venn diagram showed the number of overlapping mRNAs according to RNA-seq and targets prediction. **B** Constructing miRNA-mRNA network using degree algorithm of cytoHubba. **C** Protein–protein interaction (PPI) network analysis of 113 differential mRNAs. **D** Construction of miRNA-mRNA networks according to these overlapping mRNAs enriched in oxidoreductase activity, apoptosis, autophagy, etc. The red color represents up-regulation and blue color represents down-regulation. **E** and **F** Kyoto Encyclopedia of Genes and Genomes (KEGG) enrichment analysis of these overlapping mRNAs. Sankey relationship diagram of these signaling pathways and mRNAs. **G** Using the random forest model performed prediction analysis. **H** Correlation analysis between GSH-Px and these gene expression, including *MAPK10*, *TRAM1L1*, *FBXO32* and *IRS4*

For revealing the key miRNA-mRNA axis influencing embryonic oxidative damage, comprehensive analysis with these DE-miRNAs and DE-mRNAs was performed. A total of 113 mRNAs were identified, in which 224 miRNA-mRNA networks within *r* < −0.6 and *P* < 0.05 were constructed using the degree algorithm of cytoHubba (Fig. 5A and B). With interaction score > 0.4, there were 32 genes with significant interrelationship (Fig. 5C). *MAPK10* and *HSD3B1* present higher interaction score and more interactive networks (Fig. 5C). On this foundation, miRNAs and mRNAs involved into cellular process and redox regulation were selected to exhibit further analysis. miRNA-mRNA networks were constructed, including 11 mRNAs, 10 miRNAs and 28 miRNA-mRNA pairs (Fig. 5D). According to KEGG pathway analysis, most of those genes were mainly involved in programmed cell death, as evidenced by the lower *P*-value and more abundant genes in autophagy, apoptosis and necroptosis (Fig. 5E and F). Subsequently, random forest model and correlation analysis were performed for disclosing the key miRNA-mRNA axis. According to ROC curve results, the AUC values of these genes were all over than 75%, in which *MAPK10*, *TRAM1L1*, *FBXO32* and *IRS4* had AUC values of 100%, 94%, 94% and 92% (Fig. 5G). Meanwhile, expression of *MAPK10*, *TRAM1L1*, *FBXO32* and *IRS4* were significantly related with activities of GHS-Px (Fig. 5H). *MAPK10* might be the crucial target gene for these miRNAs to function, taking into account the highest Matthews correlation coefficient (MCC) and area under the ROC curve (AUC) value, fold change and correlation with anti-oxidant index (Fig. 5G and 3E). miR-145-5p with highest fold change was predicted as the important epigenetic regulator of *MAPK10*, and their target genes were mainly enriched into apoptosis and MAPK signaling pathway (Fig. 5D and S4E). Thus, miR-145-5p/*MAPK10* axis might play significant roles in oxidative damage of chicken embryonic liver.

### miR-145 alleviates H_2_O_2_-induced hepatocyte apoptosis by targeting *MAPK10*

**Fig. 6:**
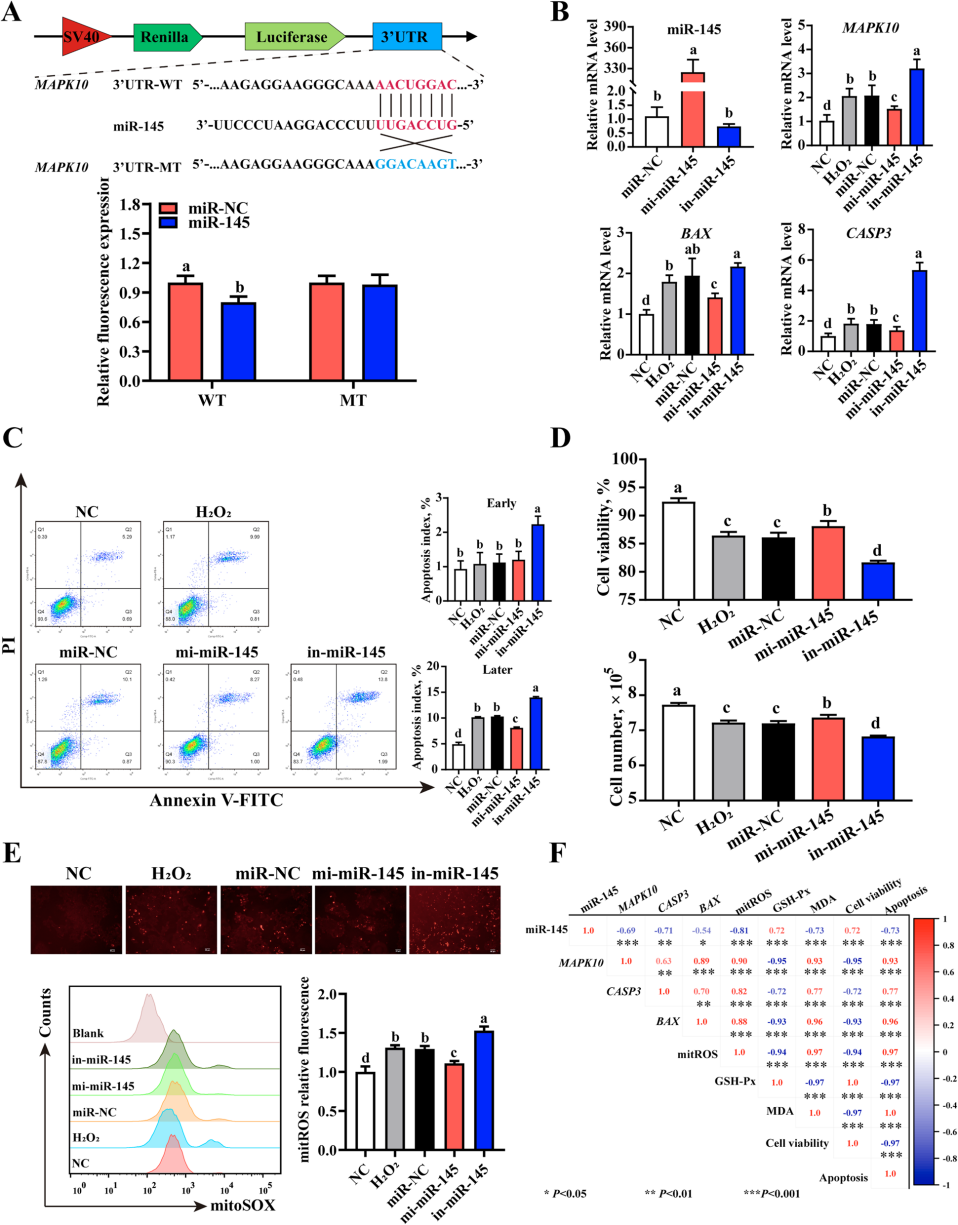
Alleviation of miR-145 on H_2_O_2_-induced hepatocyte apoptosis. **A** Schematic diagram of miR-145 targeting *MAPK10*. Targeting relationship between miR-145 and *MAPK10* was measured by co-transfection with miR-145 mimics and wild-type or mutant-type (WT or MT) dual-luciferase reporter gene. **B** Expression of miR-145, *MAPK10*, *CASP3* and *BAX* in hepatocytes. **C** and **D** Analysis of cell viability, cell number and apoptosis index. **E** Mitochondrial superoxide in hepatocytes was detected using MitoSOX™ Red by flow cytometry, fluorescence microscope and microplate reader. **F** Overview of correlation analysis. ^a–d^The different letters represent significant difference (*P* < 0.05). ^*^P < 0.05, ^**^P < 0.01. NC and H_2_O_2_ mean hepatocytes without any treatment and with H_2_O_2_ treatment separately; miR-NC (negative control fragments), mi-miR-145 (mimics of miR-145) and in-miR-145 (inhibitors of miR-145) mean hepatocytes with H_2_O_2_ treatment following transfection of control sequence, miR-145 mimics or inhibitors separately

For verifying the targeting relationship between miR-145 and *MAPK10*, bioinformatics prediction and dual luciferase reporter experiment were performed (Fig. 6A). As shown in Fig. 6A, transfection of miR-145 decreased the relative luciferase activity of pmirGLO-*MAPK10*-wt, but unchanged the relative luciferase activity of pmirGLO-*MAPK10*-mt, implying that miR-145 could target *MAPK10* to function. Over-expression of miR-145 could be achieved with mimic transfection (Fig. 6B). Meanwhile, over-expression of miR-145 significantly inhibited H_2_O_2_-stimulated *MAPK10*, *CASP3* and *BAX* up-regulation, whereas inhibition of miR-145 markedly aggravated *MAPK10* and *CASP3* up-regulation (Fig. 6B). Compared to miR-NC group, miR-145 transfection critically increased cell viability and attenuated apoptosis (Fig. 6C and D). H_2_O_2_-induced MDA and mitochondrial ROS (mitoROS) level increase were also inhibited with miR-145 transfection (Fig. 6E and S5). According to correlation analysis, over-expression of miR-145 was positively related to cell viability and negatively related to apoptosis, mitoROS level and *MAPK10*, *CASP3* and *BAX* expression (Fig. 6F). In summary, miR-145 possibly targeted MAPK10 and decreased mitoROS level, attenuating H_2_O_2_-stimulated hepatocyte apoptosis.

## Discussion

Egg exosome miRNAs possibly involved in modulating embryonic oxidation–reduction process, but the key miRNA and the specific regulative mechanism still require experimental evidence [11]. In this study, differentially expressed miRNAs in egg exosomes could exacerbate hepatocyte apoptosis and peroxide generation by their target mRNAs, inducing or aggravating liver oxidative damage of chick embryos in aged breeding hens. In addition, exosome miR-145-5p could target *MAPK10* and hinder mitoROS accumulation and overexpression of *BAX* and *CASP3*, attenuating H_2_O_2_-induced hepatocyte apoptosis in chick embryos. These findings reveal a novel target for protecting embryonic liver against oxidative damage to maintain embryonic redox homeostasis.

The declined hatching performances in aged breeding hens has been reported extensively, such as the decreased hatchability and day-old chick weight and increased embryonic mortality and malformation [2, 35–37]. It was also observed as the decreased hatchability and chick quality in 65-week-old breeding hens compared to 36-week-old breeding hens. While the significant difference was not fond in chick weight between the two groups. It was found that body weight of hatched chicks was positively related to the initial egg weight [37]. Therefore, the similar body weight of chicks in the two groups might be attributed to that the initial egg weight was not significant differences. In addition, embryonic mortality in the late stage of incubation was significantly elevated in 65-week group, which was consistent with previous observation [2]. The increased embryonic mortality during the late stage of incubation might be mainly attributed to the irreversible oxidative damage induced by free radicals and peroxide agents [38]. For aged breeding hens, a large number of pro-oxidant molecules could be accumulated into fertile eggs, and in turn aggravating the peroxide production and accumulation in chick embryos [5–7]. Similarly, the compromised antioxidant system and increased peroxide agents in 65-Em at E18 were shown as the increased MDA levels and decreased SOD, GSH-Px and CAT activities. As the hub organ of sustaining redox balance, insufficient function of liver in clearing free radicals and peroxides could lead to oxidative damage of organism and even inducing disease and death [39–41]. In the present study, the increased embryonic mortality in 65-week-old breeding hens might be attributed to the elevated peroxide agents in embryonic liver, supported by the significant correlation between embryonic mortality and GSH-Px activity and MDA level. Thus, these findings suggested that peroxide accumulation might cause oxidative damage of chick embryos, contributing to the higher embryonic mortality in aged breeding hens.

For disclosing the specific reason of oxidative imbalance in 65-Em liver in depth, we performed transcriptomic and bio-informatics analysis to explore the differential mRNAs expression and critical signaling pathways. In the present study, DEGs were significantly enriched into oxidoreductase activity and glutathione metabolism pathways. These signaling pathways exhibited important regulative effects for synthesis and function of endogenous antioxidants such as glutathione, SOD and GSH-Px [42, 43]. It was implied that differential expression of these genes might be responsible for the compromised antioxidant system in chick embryos’ liver. In chick embryos, GSH-Px serves as the predominant antioxidant enzyme, with its demand in embryonic liver exhibiting a significant upregulation during late incubation stages. [7]. Here, the lower GSH-Px activity has been found to associate with the elevated *MAPK10*, *ADAM20*, *KCNA5* and *TFAP2C* expression and the decreased *VAT1L*, *FGF7* and *FMOD* expression in embryonic liver, which is consistent with previous reports [44–50]. Thus, these findings demonstrated that the perturbation of DEGs might destroy antioxidant system and cause peroxide accumulation in 65-Em liver. Excessive accumulation of peroxide agents easily induced oxidative stress, injuring cellular structure and triggering programmed cell death [51]. Excessive cell death, in turn, could exacerbate oxidative stress, forming a vicious cycle that results in irreversible oxidative damage, disease and even death to animals [52–55]. In the present study, most of DEGs were enriched into apoptosis signal pathway. Meanwhile, the majority of DEGs in this pathway exhibited significant correlation with GSH-Px activity, MDA levels and embryonic mortality. These results in this study suggested that genes exacerbating peroxide accumulation and apoptosis might be responsible for oxidative damage aggravation in 65-Em liver.

Exosome miRNAs from maternal sources are a type of important genetic biomolecules, which can exhibit pivotal effects in embryonic development as well as physiological and pathological processes by inhibiting transcription and translation of key genes [56, 57]. Our previous study had shown that miRNAs in egg exosomes could participate in oxidation–reduction processes and apoptosis, hinting that egg exosome miRNAs might perform important effects in regulating oxidative damage of chick embryos [11]. In the present study, we identified 13 DE-miRNAs within exosomes derived from 65-Eg and 36-Eg. Bioinformatics analysis revealed that target genes of DE-miRNAs were significantly enriched in signaling pathways that regulate redox balance and programmed cell death, such as oxidoreductase activity, cellular senescence, autophagy and apoptosis, which were consistent with observations in transcriptomic analysis. Meanwhile, the majority of DEGs were predicted to be target genes of DE-miRNAs. It seemed to support the hypothesis that exosome DE-miRNA could result in the differential expression of genes that identified in embryonic liver. In addition, integrative analysis shown that target mRNAs of miRNAs were mainly enriched in apoptosis signal pathway. Most of these target mRNAs were significantly correlated with embryonic mortality, GSH-Px activity and MDA levels. It means that exosome miRNAs could target those DEGs in embryonic liver to exacerbate hepatocyte apoptosis and peroxide generation, inducing or aggravating liver oxidative damage of chick embryos in aged breeding hens.

Comprehensive analysis was carried out to identify the key miRNA-mRNA axis that performed alleviation in hepatic oxidative damage of chick embryos. In the present study, *MAPK10* was found to be the hub gene and might aggravate hepatocyte death and ROS accumulation in embryonic liver, evidenced by the highest MCC and AUC values and KEGG enrichment analysis. Meanwhile, miR-145-5p with highest fold change was the important epigenetic regulator of *MAPK10*, and their target genes were mainly enriched into apoptosis and MAPK signaling pathway. Thus, it could be inferred that miR-145-5p/*MAPK10* axis might exhibit vital regulatory effects in oxidative damage of embryonic liver. Several reports suggested that miR-145 could ameliorate oxidative stress-induced cell apoptosis and inflammatory damage through improving antioxidant system and suppressing generation of pro-oxidant molecules [58, 59]. Taken together, these findings indicated that miR-145-5p possibly targeted *MAPK10* inhibiting oxidative stress-induced excessive apoptosis in chicken embryonic hepatocytes, but the specific effect and mechanism await further research.

Given that miR-145-5p/*MAPK10* axis might alleviate oxidative stress-induced hepatocyte apoptosis, the specific role and mechanism were investigated using H_2_O_2_-challenged primary hepatocytes of chick embryos. In the current study, H_2_O_2_ administration elevated apoptotic rate and *CASP3* and *BAX* expression, but these results were reversed following the transfection of miR-145-5p. It implies that over-expression of miR-145-5p could attenuate oxidative stress induced apoptosis in embryonic hepatocytes. These findings are similar with previous reports on mouse in vivo or in vitro [58, 59]. Notably, *MAPK10*, up-regulated in H_2_O_2_-stimulated hepatocytes and negatively regulated by miR-145-5p, was demonstrated as the direct target of miR-145-5p, based on correlation analysis, bioinformatics prediction and dual-luciferase reporter assay. *MAPK10* is the member of JNK subgroup of the mitogen-activated protein kinase, which has been demonstrated to be the critical integration point for multiple signaling pathways and play key roles in diversified physiological processes such as apoptosis and proliferation [59]. It was found that *MAPK10* up-regulation could activate transcription factors such as c-Jun and c-Fos, promoting Bax and Caspase-3 expression as well as inhibiting Bcl-2 expression, and in turn exerting pro apoptosis by a series of cascade reactions [60, 61]. In mice, the down-regulation or deficiency of *MAPK10* resulted in the decreased Caspase-3 and Bax expression, attenuating cell apoptosis [62]. In the present study, apoptotic index as well as *CASP3* and *BAX* levels showed a significantly positive correlation with *MAPK10* expression and noticeably negative correlation with miR-145-5p expression. Hence, these findings indicated that miR-145-5p could target *MAPK10* to inhibit *BAX* and *CASP3* expression, alleviating H_2_O_2_-challenged apoptosis in chicken embryonic hepatocytes. ROS, primarily produced in mitochondria, are critical signaling molecule that maintain redox balance and act as key regulators of oxidative damage and cellular apoptosis [63]. Overload of mitoROS could injure mitochondrial biomolecules and destroy mitochondrial structure and function, activating Bax/Caspase-3 axis to promote apoptosis [64]. When escaping into cytoplasm, mitoROS would activate p53 and JNK signaling pathways further elevating Bax and Caspase-3 expression [65]. In the current study, our results showed that miR-145-5p transfection inhibited H_2_O_2_-stimulated mitoROS increases. Meanwhile, mitoROS levels were critically positively related to *MAPK10*, *BAX* and *CASP3* expression. It was prompted that miR-145-5p could target *MAPK10* to hinder mitoROS accumulation, attenuating H_2_O_2_-induced hepatocyte apoptosis in chick embryos. Collectively, these findings might provide a potential target for attenuating oxidative damage of chicken embryonic liver.

## Conclusions

In conclusion, egg exosome miRNAs could exacerbate oxidative damage in embryonic liver, contributing to the increased embryonic mortality in aged breeding hens. miRNA-mRNA network analysis revealed the potential roles of miR-145-5p/*MAPK10* axis in alleviating embryonic hepatocyte apoptosis and peroxide accumulation. Furthermore, we found that miR-145-5p could target *MAPK10* and inhibit mitoROS accumulation, attenuating H_2_O_2_-induced hepatocyte apoptosis in chick embryos. These findings reveal a novel target alleviating oxidative damage of embryonic hepatocytes, which provide theoretical basis and guidance for supporting fetal health and development by improving maternal physiological status through nutritional or genetic modifications.

## Supplementary Information

Additional file 1: Table S1. PCR primer specifications. Table S2. Gene Ontology (GO) functional enrichment analysis. Figure S1. Physical parameters of fertilized eggs in 36 and 65 week-old breeding hens. Figure S2. Differential analysis of embryonic serum and muscle antioxidant capacities. Figure S3. Analysis of features of differentially expressed mRNAs in embryonic liver. Figure S4. Analysis of features of differentially expressed miRNAs. Figure S5. SOD, CAT and GSH-Px activities and MDA levels in H_2_O_2_ induced primary hepatocytes

## Data Availability

Datasets used in the present study are available from the corresponding author on request.

## Abbreviations

36-Eg: Eggs from 36-week-old breeder flocks
65-Eg: Eggs from 65-week-old breeder flocks
36-Em: Embryos from 36-Eg
65-Em: Embryos from 65-Eg
AUC: Area under the ROC curve
CPM: Counts Per Million
E18: 18^th^ d of incubation
E19: 19^th^ d of incubation
MCC: Matthews correlation coefficient
ROS: Reactive oxygen species
