# Supplementary material for: Egg exosome miR-145-5p decreases mitochondrial ROS to protect chicken embryo hepatocytes against apoptosis through targeting MAPK10
Source: J Anim Sci Biotechnol. 2025 May 24;16:74. doi: 10.1186/s40104-025-01203-y (PMC12103047; doi:10.1186/s40104-025-01203-y)
Supplement: Supplementary file 1 — Additional file 1: Table S1. PCR primer specifications. Table S2. Gene Ontology (GO) functional enrichment analysis. Figure S1. Physical parameters of fertilized eggs in 36 and 65 week-old breeding hens. Figure S2. Differential analysis of embryonic serum and muscle antioxidant capacities. Figure S3. Analysis of features of differentially expressed mRNAs in embryonic liver. Figure S4. Analysis of features of differentially expressed miRNAs. Figure S5. SOD, CAT and GSH-Px activities and MDA levels in H2O2 induced primary hepatocytes [file 40104_2025_1203_MOESM1_ESM.docx]

**Supplemental tables and figures**

**Table S1** PCR primer specifications

| **Gene** | **Primer sequence** |
| --- | --- |
| *GAPDH* | F: 5′-GGTGCTAAGCGTGTTATCATCTCA-3′ |
|  | R: 5′-CATGGTTGACACCCATCACA-3′ |
| *BAX* | F: 5′-ACAGGGTTTCATCCAGGATCGAGCA-3′ |
|  | R: 5′-TCAGCTTCTTGGTGGACGCATC-3′ |
| *CASP3* | F: 5′-TGGCCCTCTTGAACTGAAAG-3′ |
|  | R: 5′-TCCACTGTCTGCTTCAATACC-3′ |
| *MAPK10* | F: 5′-CACGGTTGACCTCTGAGCTT-3′ |
|  | F: 5′-TGGCAAGGTGAACAGTTGGT-3′ |
| *TBX18* | F: 5′-ACTAGCCAGCTCTGTAGCCT-3′ |
|  | R: 5′-GAATGCATCCCCGTCATTGC-3′ |
| *FGF7* | F: 5′-GTGAACTGTTCCAGCCCTGA-3′ |
|  | R: 5′-TGCCACTATTCCAACTGCCA-3′ |
| *TMEM178B* | F: 5′-GGGATCGAGGCCTTATGCAA-3′ |
|  | R: 5′-GACAGGTTGGACAGAAGGGG-3′ |
| *FMOD* | F: 5′-GACCCACCCGGGTTTAACAT-3′ |
|  | R: 5′-TGCTGCCCATGCAGACATTA-3′ |
| *SLC15A2* | F: 5′-AGGTTTAGGCTGTGCTCCAC-3′ |
|  | R: 5′-CGTGGTGAGTTGACTGGGAA-3′ |
| *CAPN2* | F: 5′-TGGCAGAAGAATCTGCAACCT-3′ |
|  | R: 5′-GGACCTTGTGGTGCTGCTAT-3′ |
| *GHSR* | F: 5′-TGCCTTTTCACGTAGGACGA-3′ |
|  | R: 5′-GCGCTCAGGTAGAAGAGGAC-3′ |
| *ID4* | F: 5′-CGACTACATCCTCGACCTGC-3′ |
|  | R: 5′-CTTGTTAACAGAGCCAGCCG-3′ |
| *FST* | F: 5′-TCACGCTGTTCCTGTGTCTC-3′ |
|  | R: 5′-GTTGTCGTTGACGTCCTCCT-3′ |
| *HSD3B1* | F: 5′-TGTTTAGCACTGAGGCAAGAG-3′ |
|  | R: 5′-CCTCAGTCTTGCCCTGGAAC-3′ |
| *BCL2A1* | F: 5′-AAGCTTCAAGAGCACGGAGT-3′ |
|  | R: 5′-GTTTTCCCAGCCACCGTTTG-3′ |
| *CYP2AC1* | F: 5′-TGTTTCCCATCCTTGGACCC-3′ |
|  | R: 5′-TGGCCTTTTCATTCTGCTCTT-3′ |
| *TUBA1A* | F: 5′-GCTGCTCGTACAGGGAACAA-3′ |
|  | R: 5′-TGAGACACACAGGAGAGGACA-3′ |
| *MAP2K2* | F: 5′-TGCTGATGAATCACACCTTCAT-3′ |
|  | R: 5′-TGCTGGGCTGGTTTAACCTC-3′ |
| *CYCS* | F: 5′-CGGCCGGCTAAGCAGTA-3′ |
|  | R: 5′-TGGGTCCAGTCTTGTGCTTG-3′ |
| *GLUL* | F: 5′-GGGAGCCGAGCGTGG-3′ |
|  | R: 5′-CAGTCCCGTCGATCCAGATG-3′ |
| *MGST3* | F: 5′-CAGCGGTGCCATAGCGAG-3′ |
|  | R: 5′-TACTTCTTGCGGGCCTTTCC-3′ |
| *ALKBH2* | F: 5′-CAGGAGAGTGCTTGCTCCAA-3′ |
|  | R: 5′-CCTTGGATCATCAAACGCAGC-3′ |
| *DTL* | F: 5′-GTCCAGACGACCAGTTCCTG-3′ |
|  | R: 5′-GTAGGCGCCAGATCCTTACC-3′ |
| *AvBD88* | F: 5′-CATGCGCGTACCTAACAACG-3′ |
|  | R: 5′-CTGAGGTCCTGGCGAACATT-3′ |
| *SSX2IP* | F: 5′-CGGTCCCTCCTCCCTTCT-3′ |
|  | R: 5′-GTACCTGAGCAACCCGTTCA-3′ |
| *CDX4* | F: 5′-TGAGGAAAACGGTGCCAACT-3′ |
|  | R: 5′-GGCTGAGTGAACCAGAGTCA-3′ |

**Table S2** Gene ontology (GO) functional enrichment analysis

| **KO ID** | **Description** |
| --- | --- |
| **Biological Process** | |
| GO:0045944 | positive regulation of transcription by RNA polymerase II |
| GO:0006355 | regulation of DNA-templated transcription |
| GO:0007165 | signal transduction |
| GO:0006468 | protein phosphorylation |
| GO:0000122 | negative regulation of transcription by RNA polymerase II |
| GO:0045893 | positive regulation of DNA-templated transcription |
| GO:0006357 | regulation of transcription by RNA polymerase II |
| GO:0007186 | G protein-coupled receptor signaling pathway |
| GO:0055085 | transmembrane transport |
| GO:0043066 | negative regulation of apoptotic process |
| GO:0008284 | positive regulation of cell population proliferation |
| GO:0035556 | intracellular signal transduction |
| GO:0010628 | positive regulation of gene expression |
| GO:0006811 | monoatomic ion transport |
| GO:0043065 | positive regulation of apoptotic process |
| GO:0045892 | negative regulation of DNA-templated transcription |
| GO:0006508 | proteolysis |
| GO:0008285 | negative regulation of cell population proliferation |
| GO:0006470 | protein dephosphorylation |
| GO:0016567 | protein ubiquitination |
| GO:0006974 | DNA damage response |
| GO:0015031 | protein transport |
| GO:0007155 | cell adhesion |
| GO:0006886 | intracellular protein transport |
| GO:0006511 | ubiquitin-dependent protein catabolic process |
| GO:0030154 | cell differentiation |
| GO:0010629 | negative regulation of gene expression |
| GO:0006629 | lipid metabolic process |
| **Molecular Function** | |
| GO:0008270 | zinc ion binding |
| GO:0005509 | calcium ion binding |
| GO:0000978 | RNA polymerase II cis-regulatory region sequence-specific DNA binding |
| GO:0004672 | protein kinase activity |
| GO:0005515 | protein binding |
| GO:0042802 | identical protein binding |
| GO:0005524 | ATP binding |
| GO:0003677 | DNA binding |
| GO:0046872 | metal ion binding |
| GO:0042803 | protein homodimerization activity |
| GO:0003700 | DNA-binding transcription factor activity |
| GO:0003676 | nucleic acid binding |
| GO:0005525 | GTP binding |
| GO:0003723 | RNA binding |
| GO:0000166 | nucleotide binding |
| GO:0043565 | sequence-specific DNA binding |
| GO:0019901 | protein kinase binding |
| GO:0003924 | GTPase activity |
| GO:0016787 | hydrolase activity |
| GO:0000981 | DNA-binding transcription factor activity, RNA polymerase II-specific |
| GO:0003824 | catalytic activity |
| GO:1990837 | sequence-specific double-stranded DNA binding |
| GO:0016491 | oxidoreductase activity |
| GO:0004930 | G protein-coupled receptor activity |
| GO:0016740 | transferase activity |
| GO:0003682 | chromatin binding |
| GO:0001228 | DNA-binding transcription activator activity, RNA polymerase II-specific |
| GO:0019899 | enzyme binding |
| **Cellular Component** | |
| GO:0005634 | nucleus |
| GO:0005737 | cytoplasm |
| GO:0016020 | membrane |
| GO:0005829 | cytosol |
| GO:0005654 | nucleoplasm |
| GO:0016021 | membrane |
| GO:0005886 | plasma membrane |
| GO:0005739 | mitochondrion |
| GO:0005783 | endoplasmic reticulum |
| GO:0005794 | Golgi apparatus |
| GO:0043231 | intracellular membrane-bounded organelle |
| GO:0005730 | nucleolus |
| GO:0005615 | extracellular space |
| GO:0032991 | protein-containing complex |
| GO:0005576 | extracellular region |
| GO:0005887 | plasma membrane |
| GO:0005813 | centrosome |
| GO:0048471 | perinuclear region of cytoplasm |
| GO:0005789 | endoplasmic reticulum membrane |
| GO:0009986 | cell surface |
| GO:0005768 | endosome |
| GO:0005764 | lysosome |
| GO:0005856 | cytoskeleton |
| GO:0016607 | nuclear speck |
| GO:0000785 | chromatin |
| GO:0098978 | glutamatergic synapse |
| GO:0000139 | Golgi membrane |
| GO:0031410 | cytoplasmic vesicle |

| **Fig. S1** Physical parameters of fertilized eggs in 36-week and 65-week old breeder hens. (A) Wet weight of eggs, albumen and yolk. (B) Dry weight of albumen and yolk, as well as the yolk:albumen ratio. Note: The asterisk represents significant difference (**P <* 0.05; ***P <* 0.01)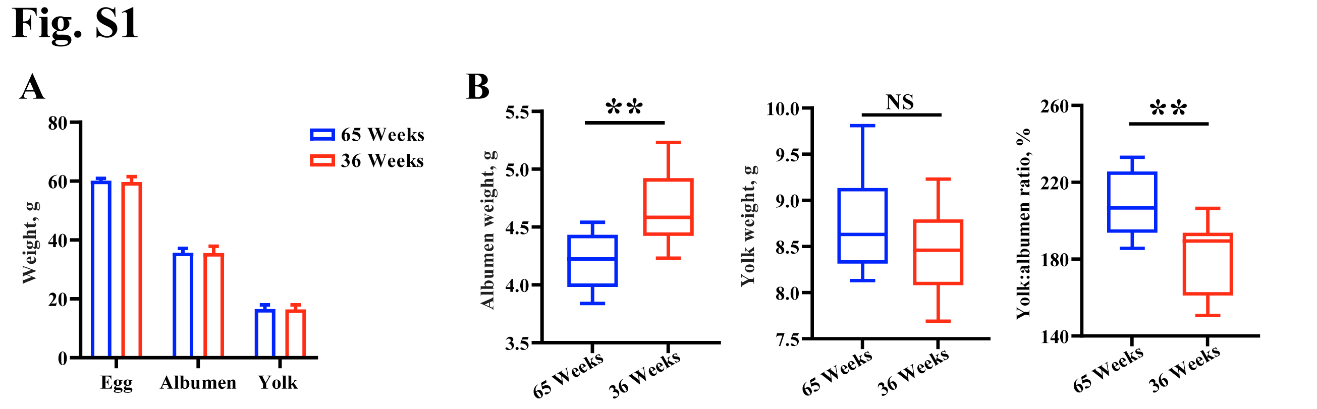 |
| --- |
|  |

| \| 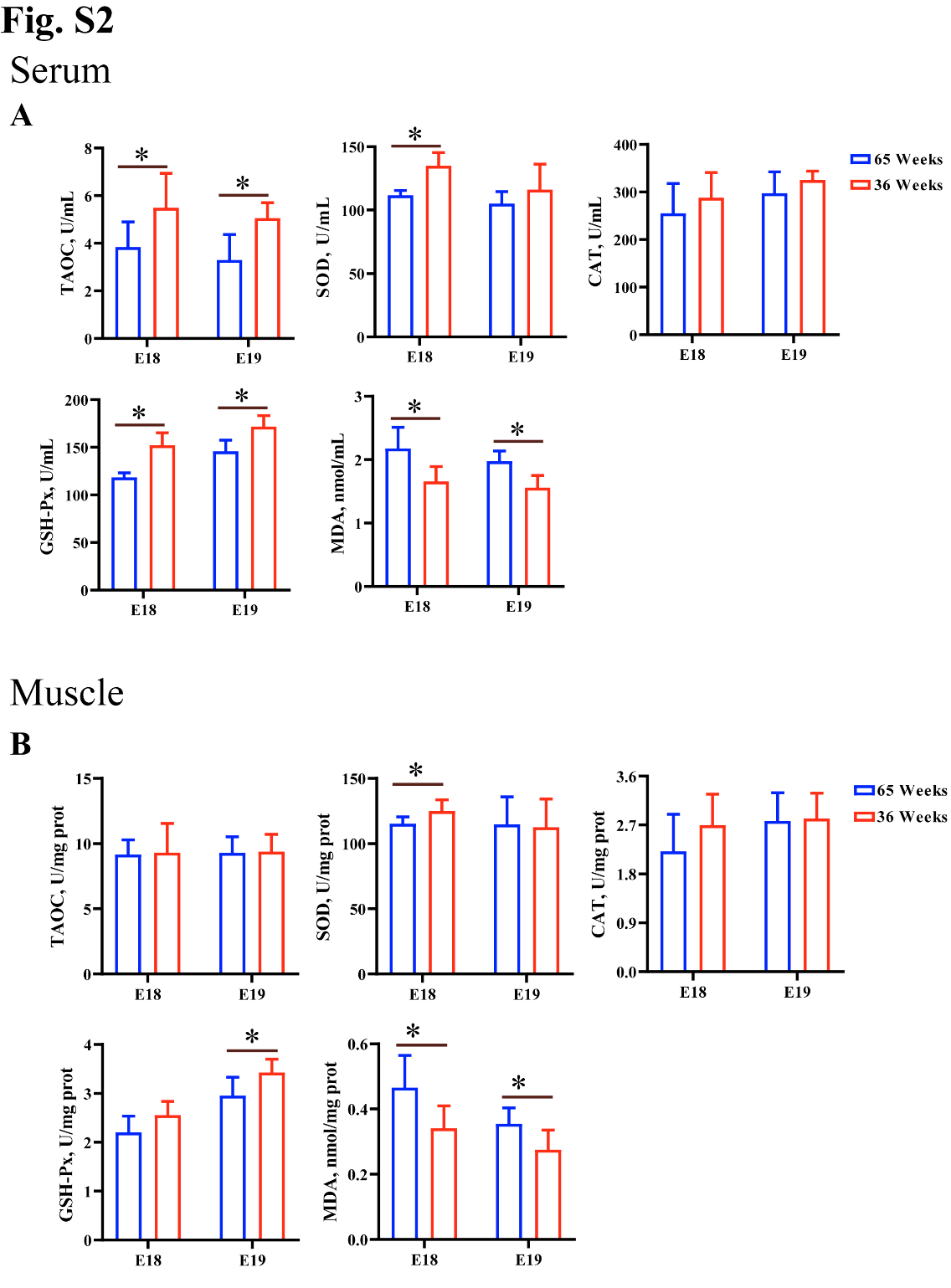 \| \| --- \| \| **Fig. S2** Differential analysis of embryonic serum and muscle antioxidant capacities. (A, B) T-AOC, SOD, CAT and GSH-Px activities and MDA levels in serum and muscle of embryos at E18 and E19. Note: The asterisk represents significant difference (**P <* 0.05; ***P <* 0.01) \| |
| --- | --- | --- |
|  |

| 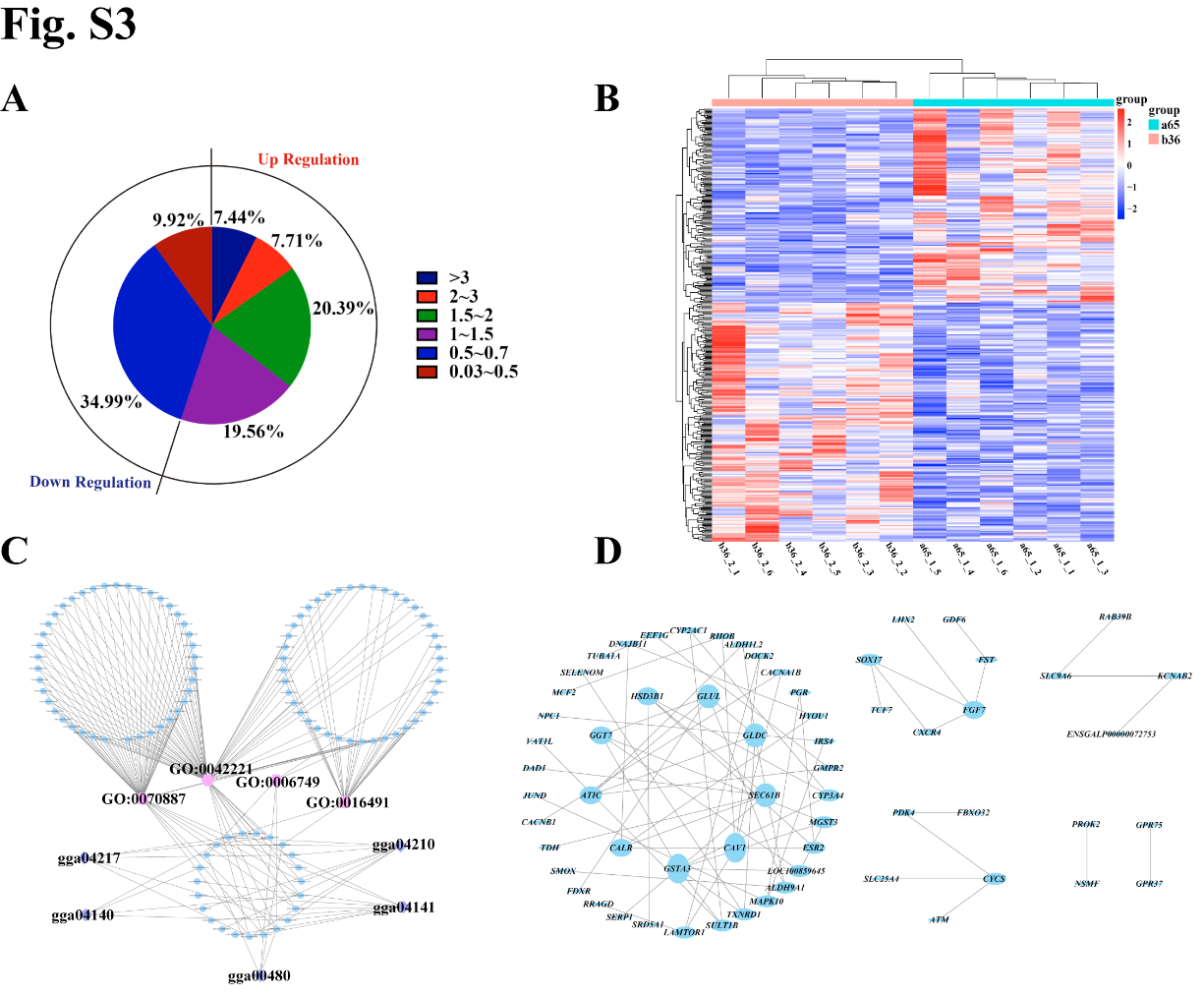 |
| --- |
| **Fig. S3** Analysis of features of differentially expressed mRNAs in embryonic liver. (A) Fold change distribution of 363 differentially expressed mRNAs. (B) Heatmap of differentially expressed mRNAs. (C) Regulatory networks constructed with mRNAs enriched in signal pathways relating to redox balance. (D) Network diagram of protein-protein interaction analysis |

| 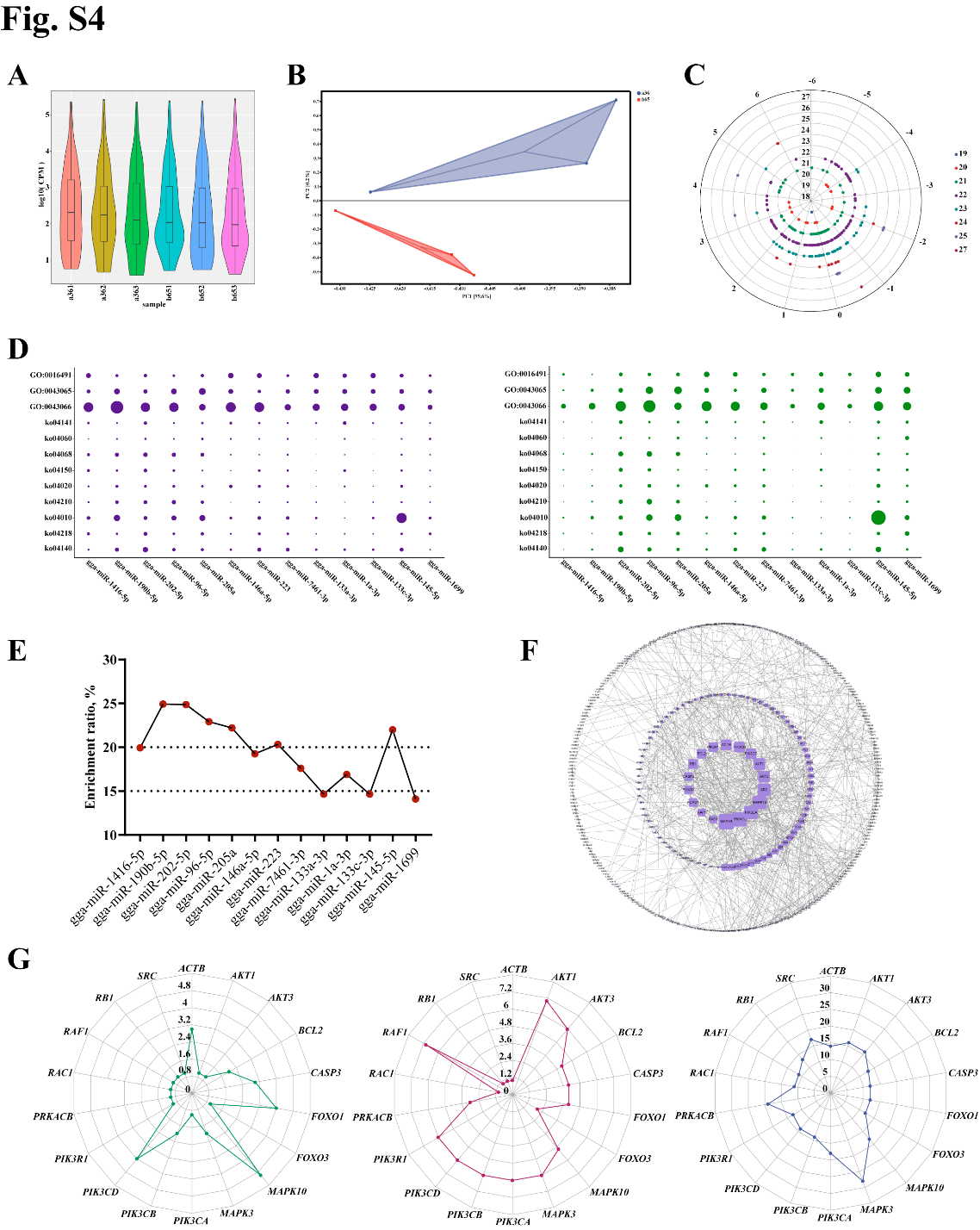 |  |
| --- | --- |
| **Fig. S4** Analysis of features of differentially expressed miRNAs. (A) Violin plots of expression levels of miRNAs. (B) Principal component analysis (PCA) of differentially expressed miRNAs. (C) Length distribution of miRNAs. (D, E) Distribution and enrichment rate of target genes of miRNAs in redox balance related pathways. (F) Network diagram of these target genes constructed by protein-protein interaction analysis. (G) Radar map performed the distribution of 18 key mRNAs in related signaling pathways and protein-protein interaction networks |  |
| 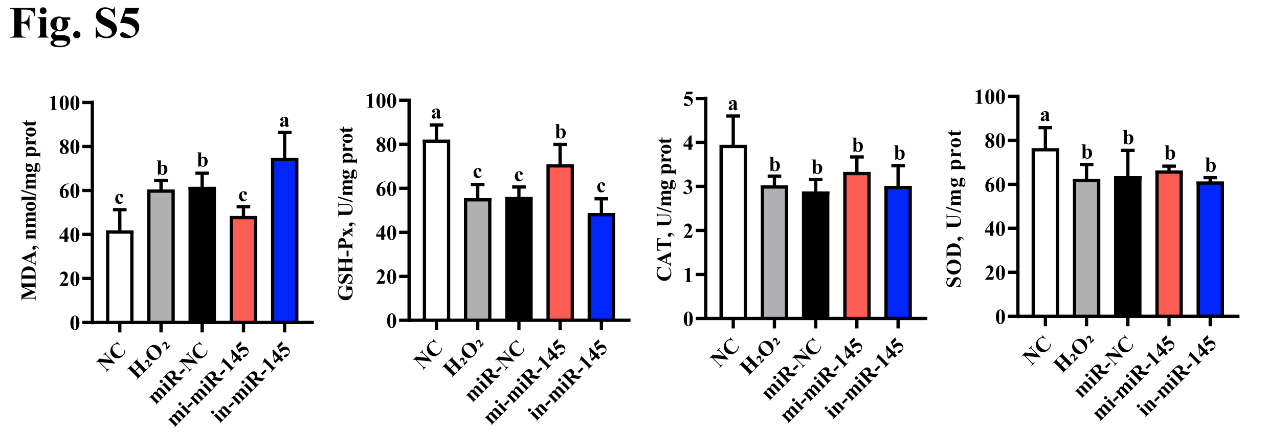 | |
| **Fig. S5** SOD, CAT and GSH-Px activities and MDA levels in H_2_O_2_ induced primary hepatocytes. The different letters represent significant difference (*P<* 0.05) | |
